# Celebrities and spiritual gurus: Comparing two biographical accounts of kidney transplantation and recovery

**DOI:** 10.4102/ajod.v4i1.151

**Published:** 2015-05-08

**Authors:** Rose Richards

**Affiliations:** 1 Research and Writing Laboratory, Language Centre, Stellenbosch University, South Africa; 2 Department of Psychology, Stellenbosch University, South Africa

## Abstract

**Background:**

As a kidney transplant recipient I have long been exposed to a shortage of renal narratives and to a dominant theme in those that exist: transplant as restitution or redemption. My lived experience has, however, shown me that post-transplant life is more complex. Even after transplantation, chronic kidney disease requires lifelong health care with varying degrees of impairment, resulting in ongoing liminality for those who experience it. Nonetheless, as a transplant recipient I find the restitution or redemptive narrative pervasive and difficult to escape.

**Objective:**

I examined two seemingly very dissimilar insider renal biographies, Janet Hermans's *Perfect match: A kidney transplant reveals the ultimate second chance*, and Steven Cojocaru's *Glamour, interrupted: How I became the best-dressed patient in Hollywood*, to explore how the narrators treat chronic kidney disease and transplantation.

**Methods:**

In addition to a close textual reading of the biographies, I used my own experience of meaning-making to problematize concepts around restitution or redemptive narratives.

**Results:**

I found that the two biographies are, despite appearances and despite the attempts of one author to escape the redemptive form, very much the same type of narrative. The accounts end with the transplant, as is common, but the recipients’ lives continue after this, as they learn to live with their transplants, and this is not addressed.

**Conclusions:**

Emphasising restitution or redemption might prevent an understanding of post-transplant liminality that has unique characteristics. The narrator evading this narrative form must come to terms with a changed identity and, sometimes, fight to evade the pervasive narratives others impose.

## Introduction

Although there are approximately 45 000 kidney transplants performed a year in the world, making kidney transplants the most common solid organ transplant (Davids, Marais & Jacobs 2014; Helman 2007:42), there are few published insider biographies on end stage renal disease (ESRD), transplantation and recovery. This is possibly because some diseases have a dimension of a social identity and metaphorical significance (Couser 1997; Sontag 2001), whilst kidney failure does not carry the same burden of meaning. Indeed there is little symbolic value to kidney failure. If an illness carries a metaphorical resonance, people who do not suffer from it can identify with its concerns. If it does not, they cannot that easily do so. Kidney failure has the same mortality rate as some forms of cancer and the same success and failure rate post-transplant as many cancers have after radiation therapy (Davids, Marais & Jacobs 2014; National Kidney Disease Education Program 2005), but its name does not strike the same chord of fear as cancer's does.^1^ As a result kidney failure is rendered less visible in society than, for instance, cancer and therefore has an unusual liminal dimension with which to contend.

When people do write about their experience of renal disease they tend to adapt their accounts to fit conventional narrative forms. One such form is the restitution narrative (Frank 1995), which attempts to derive meaning from ‘biographical disruption’ (Bury 1982). This type of narrative is a subcategory of the confessional narrative (Frank 1995). The type of restitution may take the form of a benefit (often spiritual) gained from suffering and loss. Quite easily this type of narrative may, however, develop into a type of redemptive narrative (McAdams 2006, 2008) as I shall discuss. I see redemptive narratives as essentially comic narratives that are overly simplistic when applied to life events. These types of narratives may be useful to gain a sense of control over such events but, from experience, I doubt that they help one to understand the events because redemptive narratives tend to elide ambiguity. To me it seems glib to use a narrative form that always promises some sort of closure or benefit when one's experience shows that this is not always the case. Questions that must be asked of redemptive narratives are, ‘What precisely is being redeemed?’ and ‘At what price is this redemption achieved?’

The redemptive nature of certain restitution narratives underlines their religious or moral nature. Often this type of narrative is told by an insider and alternative voice. Physical cleansing, purging and healing can easily come to symbolise social, moral or even spiritual equivalents. Illness can morph metaphorically into sin and toxins into a spiritual form of pollution (Sontag 2001). Often in the desire to fit chronic illness experiences to a narrative form and to obtain closure, a writer will try to resolve any lingering liminality. Possibly lingering liminality would undermine any attempts at closure most often associated with redemptive narratives.

Two book-length accounts of ESRD, transplantation and recovery have talked about disruption and changed identity. One is by someone who has had a transplant, Steven Cojocaru's (2007) *Glamour, interrupted: How I became the best-dressed patient in Hollywood*. The other is by a family member of someone who has had a transplant, Janet Hermans's (2006) *Perfect match: A kidney transplant reveals the ultimate second chance*. The first account is a celebrity's tale of changing body image in a world of beautiful people. The second is about kidney transplantation leading to spiritual awakening. Their differences at first seem more significant than their similarities; however, they do share certain qualities, not the least of which is that both lives are reconstructed through writing about them to derive value from the experience and to cope with ongoing liminality.

I have a particular personal interest in this because I have had a kidney transplant myself and I have finally started to write about my own experiences as part of my autoethnographic doctoral research. It has taken me more than 20 years to reach this point, because I have felt constrained not only by the overwhelming complexity of the experience, but also the narrative restrictions imposed by others on the meaning I am allowed to make. And, quite importantly, I did not previously have the verbal or emotional means to address the weirdness of liminality. Even all this time after transplant I still want to hear about other people's transplant experiences, hoping to make sense of my own life through glimpses of others’ lives (Richards 2008).

My impetus for writing as I have in this article is to see what happens when I use my own long and messy collection of experiences as a lens through which to see those of other writers. I am simultaneously insider and outsider, author and subject, researcher and researchee. Initially I sought renal biographies to teach me how to see my own. Belatedly I have started using my own experiences to help me understand others’ lives.

The two accounts I have chosen deal with the idea of disruption and damaged identity in ESRD, transplantation and recovery through the subgenre of the redemptive narrative. Looking closely at these two accounts and comparing them to my own was a surprising process. I found it difficult to make up my mind about accounts that were both like and unlike my own, paradoxically complex and simple, complete as narratives, and yet incomplete as depictions of people's lives. I have attempted to capture some of that journey here.

## Chronic illness and popular writing

At the core of the narrative paradoxes lies the problem of what to do about a serious medical condition. Serious illness and its treatments can have a profound effect on our identity, in terms of our sense of who we are individually and how we fit into the societies in which we live (Frank 1995; Kleinman 1988). This, in some sense, was at the core of my own curiosity. My illness stemmed back to early childhood, although I had my transplant in my early twenties. My kidney condition was always there, like a type of medical invisible companion or a secret sharer, part of every aspect of my life and yet seldom alluded to outside my home. My kidney disease and I developed together. It was not separate from my life ever. Once the crisis and (in my case) the transplant surgery is over, a person must return to his or her community and get on with life. But how do you go back after such devastation? And who are you afterwards?

I turned to books to find out: biography and social science. It soon became apparent to me that to understand the personal experience of illness and recovery, we need to understand the multiple contexts in which this is addressed and discussed. One such context is popular literature, which is a significant source of information in health care for lay people. This includes not only self-help books, but people trading accounts of similar experiences (Helman 2007). I noticed that whilst social scientists appreciate the importance of popular culture to lay people, they tend to treat lay people's accounts as data to support theoretical and philosophical concerns and, as a result, the accounts themselves are often derived from questions the researcher formulates (Fox & Swazey 1978; 1992; Lock 2002; Sharp 2006). An alternative is to comment on pre-existing texts, be they autobiographical or literary (Bolt 2005; Davis 1998; Freeman 1997; McLellan 1997; Minz 2001). These pre-existing accounts are typically used in discussions about illness narratives to support the theoretical and philosophical concerns of researchers.

Illness narratives that come into being from the narrator's initiative can provide researchers with pre-existing texts. They are one of the ways in which people with a common experience of illness can share information and benefit from it. Such narratives fit narrative genres that are found in other types of life writing (Frank 1995; Kleinman 1988), a significant form being the redemptive narrative, a subgenre of the confessional narrative. Whilst there are not many examples of renal biographies, I had been reading redemptive illness narratives compulsively for years. I did not realise how many I had read until I started making a list and started to see a pattern.

My pattern was fairly simple. It had two main categories. The first category was not subversive and included well-known examples of redemptive illness accounts such as Jean-Dominique Bauby's *The diving bell and the butterfly* (1997) and Anatole Broyard's *Intoxicated by my illness* (1993). These are the accounts I would feel comfortable openly discussing with other people. The second category was a type of secret, guilty pleasure. It transgressively questions and subverts the redemptive subgenre, by following the narrative trajectory for a while and then undermining it by providing a tragic ending that does not become redemptive, or by providing no closure. It surprises the reader and undermines expectations, causing one to question one's assumptions about illness narratives. Lucy Grealy's *The autobiography of a face* and Marya Hornbacher's (1999) *Wasted: A memoir of anorexia and bulimia* come immediately to mind. I felt as if the authors were speaking directly to me. I longed to find a renal biography that had the same fascination for me. The few I have found are not especially subversive.

I chose to analyse two renal biographies that were similar in certain ways. They are both book-length biographies written by insiders (a transplant recipient and the carer of a transplant recipient) for lay people, available as printed books, rather than blogs, poems or photo-essays, which are read in different ways from books. The writers come from the same culture, economic class, country and language group. The transplant recipients have both experienced long-term kidney disease and underwent transplant at roughly the same age (their thirties). This allows me to focus on a limited amount of differences. At first I was amused at the biographies’ dissimilarity. On closer inspection my amusement changed to perplexity. All was not as it seemed.

There were strange similarities in the biographies. Both were essentially redemptive after all. Although they come from very different philosophical contexts the two biographies both show the nature and persistence of liminality resulting from kidney failure and the significance of redemption for people whose identities have been damaged. Using illness narrative concepts (Frank 1995; Couser 1997) as a foundation I shall build an understanding of what liminality and redemption mean in terms of a dread disease. Then I shall look more closely at the biographies I selected to see how they treat the idea of liminality in kidney disease and how this relates to the redemptive nature of their biographies. Despite having lived through what the narrators describe in their biographies, I have to rely on other research to some extent here, because these two biographies differ from mine in one very important way. Both transplantees were well for some considerable time before becoming ill. That part of their experience does not resonate with mine.

## Damaged identities, liminality and redemption

Catastrophic illness has been described as a profound disruption of a life (Bury 1982; Jordens et al. 2001). After this, life must be re-ordered and a new meaning assigned (Kleinman 1988). Most illness narratives attempt to create meaning out of suffering in one way or the other to allow people to come to terms with profound losses (Kleinman 1988). This can be called therapeutic emplotment (Mattingly 1994). I am inclined to think that it can also be called a version of the just world hypothesis (Lerner & Miller 1978).

Sometimes the only explanation that people can come up with for such profound losses is that they must somehow have deserved them. If their accounts can show that they have made good, it can help them feel in control and perhaps they can move on. It can make them feel that the experience was not merely random, that it really does mean something and that something constructive can come of it. This also allows them to feel that the disruptive experience is over. I longed for this type of reassurance when I was waiting for my transplant. These people have ‘lived to tell the tale’ and the ritual of writing about this achievement puts it all in perspective.

However, I can vouchsafe from experience that, because a narrative is a lens, it allows us to see some things, but not others. Shared linguistic repertoires and acceptable narrative plots may serve to silence and oppress (Ezzy 1998). They also affect one's identity (Denzin 1995). Redemption through meaningful suffering and the ideal of an improved life afterwards can become more important than what happened when one was ill or the nuances of who one becomes afterwards. Sometimes illness narratives can seem to be ‘so invested in recovery’ that closure may become more important than ‘consideration of what dysfunction feels like and how it alters self-perception’ (Couser 1997:294). As meaningful as such an illness narrative might be to the narrator when first telling it, its power wanes because it is not entirely accurate. Shortly after my transplant people would often want to know what happened to me. I myself wanted to understand the extraordinary events that had so altered my life and so I told over and over the medical narrative of how I was ill, what I had suffered from, how I had got my new kidney and how well I was afterwards. And whilst telling it I felt muzzled. There were many things I could not identify, that lurked just beyond my frame of perception. There were other things I could not speak about, because it seemed like bad luck or because, as I learned early on, my audience would not want to hear about them. One of the things that slipped through the cracks was a consideration of a persistently liminal identity.

According to Van Gennep (1960) liminality is a temporary ritual state in which one withdraws from society, undergoes purification and then reintegrates into society in a new role. This reintegration is often seen as a type of rebirth. Illness can be understood as a type of liminal state because it usually requires that a person be isolated from society to be healed. Once someone has withdrawn from society, we expect them to return to society improved by their experiences, literally and figuratively, as if they had left a ritual liminal state. And yet illness is not a ritual.

The interaction between the liminal and non-liminal states in society can be seen as a relationship between anti-structure and structure (Turner 1969). This is why rituals at the end of a liminal period are so important to impose structure once again on formlessness. In modern Western society redemptive narratives might be part of our rituals of recovery that allow people who have been ill to reintegrate into society and to bring an end to their liminal state. Such narratives provide closure.

However, recovery and reintegration into society might not always be straightforward. Certain types of catastrophic illness, such as organ failure and cancer, require long-term tertiary treatment and alter one's future identity. After such a disease has rampaged through one's life one can never again be free of medical treatment and one is rendered vulnerable, because one knows it can happen again. For this reason life *after* a dread disease can also be described as a type of limbo or liminal state (Crowley-Matoka 2005; Little et al. 1998) or as ‘remission society’ (Frank 1995:8). One is usually always one or other type of patient after that, a person with ‘dual citizenship’ (Sontag 2001:3) who will ‘zigzag between the kingdom of the well and the kingdom of the ill’ indefinitely (Kolker 1996:132). People who have experienced catastrophic illness may continue to live in a state of ‘sustained’ (Little et al. 1998:1490) or ‘persistent’ (Crowley-Matoka 2005:822) liminality, sometimes for the rest of their lives.

Both renal biographies I consider are compelled to confront the issue of ongoing liminality after a transplant. They deal with this in different ways because it carries different meanings in the biographies, but the outcome is much the same because the significance of this liminality is profound to both narrators.

## The two biographies

Published biographies become published because there is a perceived readership for them. The two biographies of my title presuppose more than one type of readership. Both are aimed at readers who may undergo a transplant, or already have undergone one. They might have relevance for the family and friends of such people too. Popular culture can be a powerful resource in health care, with patients turning to other patients to draw on their experience of a disease in order to acquire knowledge about how to cope with its lived experience (Helman 2007). However the biographies are also directed at other audiences who are not renal.

The celebrity account, by Steven Cojocaru (2007), is aimed at readers who are interested in Hollywood glamour and gossip and who like to follow the lives of famous people. Such readers are keen to follow accounts that are exciting, especially if they show the traditional path of how a famous person overcame adversity. Cojocaru is a Hollywood fashion and celebrity correspondent, who has also appeared on the Oprah and Dr Phil shows. Many people would read his book because they enjoy news about celebrities and they want to know more about him.

Janet Hermans's spiritual guru biography (2006) has a specific type of religious readership in mind. The aim of her account of her husband's, Hans's, kidney transplant, is ‘to illustrate the love of Jesus and the transforming work of the Holy Spirit through the account of my husband's kidney transplant’ (Hermans 2006:xi). Its ultimate role is not to inform readers about transplantation, but to encourage them in the practice of a specific type of religious faith.

It is difficult to write an account of an illness that avoids incorporating one or other type of narrative form or bypasses any belief system. I have not yet succeeded in doing this. My medical account has developed into a dysfunctional family drama. My redemptive tale has become a quest narrative. Any meaning one derives from one's experience of illness is going to be informed by one's own values and cultural context (Toombs 1995).

Additionally, if a biography is to be published it needs to be potentially profitable and this includes having a larger, rather than a smaller, readership and using popular narrative forms. The readers’ cultural context and probable values need to be considered too. For this reason, when one relates an account of illness, one adapts it to some extent to what one imagines the hearer will want to hear (Frank 1995; Weingarten 2001). I have come to see that the audience co-constructs the meaning of the account and if the account is a confessional one, the audience is the witness. They are intrinsic to the ritual. The reintegration into society and the redemption that would result cannot happen without the one or other type of audience acknowledging the event.

Because the two accounts are so different from each other in terms of structure and intention, I shall look at them first separately and then compare some points of similarity. To allow space to explore themes I shall focus mainly on three aspects: the titles, the discovery of ESRD and life after transplant. In both cases the titles are microcosms of the accounts as wholes.

Typically in transplant accounts the life afterwards is described as an improvement on being ill (Balcita 2011; Etherington 1991; Klug & Jackson 2004) and both biographies I examine do this. In addition to this, both biographies represent life after transplant as different from life before illness and this is where the biographies take on redemptive qualities. They make sense of this in ways that may seem dissimilar at first, but that share some important similarities.

### Spiritual regeneration and ‘sins’ of omission

Janet Hermans's biography uses a parallel chapter structure to explain a religious concept through a real-life event in the form of a parable. It does this through breaking the events around Hans's transplant into eight chapters, each dealing with a different stage of the transplantation process and each followed by a chapter that handles a related theme in a Christian spiritual process and explains the connection. For example, chapter 4(a) concerns Hans's physical transformation after transplant and chapter 4(b) concerns the spiritual transformation that occurs ‘when someone experiences conversion’. Furthermore, ‘Erik [the donor] models Jesus, Erik's kidney models the Holy Spirit’ (Hermans 2006:56, 55).

Each pair of chapters is preceded by short passages from the Bible, which focuses what follows. Although the renal chapter always precedes the spiritual chapter, the Bible verses always precede the renal chapter, effectively sandwiching the renal account between biblical messages that have very clear religious intentions. This shows that the biographical part is primarily a vehicle to illustrate something spiritual. In the end, there is one overarching spiritual journey of redemption.

Hermans's title, *Perfect match: A kidney transplant reveals the ultimate second chance*, uses typical themes in transplant accounts (perfect match and second chance) to make a connection between her husband's transplant and her religious beliefs. These typical themes are significant because they influence how we portray transplantation. Transplantation is typically described as a second chance at life, which can misleadingly make it sound like a type of return or rebirth. One is not born again; instead one's life continues, although possibly not in the way one expected. From my experience, having a second chance at life can be rather burdensome and imbues even the most basic decisions with weighty importance. I feel that a ‘second chance’ implies that one's first stab at life was unsuccessful and that one is now morally obliged to make up for it as if it were one's fault – as if one were so flawed one needed to start over, whilst others were not. Receiving a perfect match can also imply a moral responsibility. If one has a second chance, one had better live up to it.

Hermans keeps her renal and religious sections in separate chapters, preventing readers from inferring that religious beliefs will cure one's kidney problems. In her renal biography, medical problems are cured by medical solutions. However, the renal sections become symbolic: the donor comes to represent Christ, the kidney the Holy Spirit and the recipient the believer accepting redemption. Toxins in the blood are equated with pollution in the soul (Hermans 2006:53) and in this light it becomes difficult to avoid seeing the sufferer as somehow deserving of sickness.

In other words, a logical leap has been made from physical problems to moral ones. It is a short step from this (albeit one that Hermans does not take) to saying that if you are ill, you have done this to yourself, because there is something spiritually wrong with you. Another theme is that of what illnesses really represent. If kidney failure represents moral turpitude, it could be very easy to see kidney failure and moral turpitude as interchangeable.

Hermans does not mention the cause of Hans's reduced kidney function, possibly because it would take on a symbolic value of sin when seen in the context of the parallel religious narrative. In the light of what the kidney represents – the Holy Spirit – in Hermans's parable (Hermans 2006:75), one might be tempted to impose a moral judgement on Hans's having flawed kidneys in the first place. Hermans occludes this deliberately, saying, ‘The details of my husband's medical record are not essential to this book’ (Hermans 2006:xi).

Hermans also does not talk about how her husband experienced the news that his kidneys were failing, because it falls outside the scope of her account which is about the possibility of salvation and the significance of donation. The renal part of her account is concise and medically oriented, so there seems to be no space for an account of ESRD to be told, only that of recovery and redemption.

This would be something very personal and subjective that she would have experienced another way, as a care provider, instead of the patient. In addition, the account of Hans's ESRD could overwhelm the account of the donor, Erik's, donation, because with chronic kidney failure people often suffer for a long time and so much relentless misery tends to leave its mark permanently. An account that included a discussion of the impact of kidney failure would, in my experience, render a narration extremely messy. For example, even all these years after transplant I still feel guilty and anxious if I eat previously ‘forbidden’ foods like chocolate or pizza. And even now I am overwhelmed with terror if I have an unexplained spike in my body temperature. Although I have a high-functioning transplanted kidney and have had it for over two decades, part of me is still living in ESRD.

It could be argued that the account of dysfunction is missing only because Hermans uses her husband's kidney transplant as a parable to illustrate a religious concept. This makes her account very focused which might arguably lead to ‘sins of omission’. However, it is important to realise that we all edit our scripts for various reasons. No account is ever complete. Her account is only about the transplant itself. What happens afterwards? If persons are redeemed through their faith, they must continue to ‘foster the Holy Spirit’ (Hermans 2006:78) as Hans must continue to look after his health.

In Hermans's account one can clearly see the theme of renewal; however it has an undercurrent of uncertainty and an implicit recognition that Hans's situation is not that of ‘normal’ good health. In fact an element of contradiction is apparent in her descriptions. ‘Hans was a new man transformed by Erik's kidney’, she says at first (Hermans 2006:53). He is no longer the old Hans, full of toxins, but a new and different person. She subsequently says, ‘My husband was restored physically’ (Hermans 2006:106). This implies that Hans returned to his pre-ESRD state, which seems at odds with her previous description and, indeed, with the religious message of her book. The uncertainty of Hans's future post-transplant is difficult for her to write about partly because it does not truly fit her parable of salvation.

This second chance is not necessarily a permanent state and to sustain it he must take his medications, eat healthily and see the doctor ‘several times a year’ (Hermans 2006:73). He has been saved, but his salvation could be taken away at any time. Hermans does not explore this, but leaves the reader with this perturbing thought in the final paragraph: ‘Hopefully Erik's kidney will see Hans through this life; we are not sure how long it will last’ (Hermans 2006:106).

Liminality deriving from an illness can be difficult and painful to articulate because it is not neat and tidy, nor is its persistence dependent upon one's behaviour. I see Hermans understanding this through her faith and the ‘passage quality of religious life’ (Turner 1969:107). If, in one's spiritual belief system life itself is seen as a liminal phase before heaven or hell, that may make it easier to understand life after catastrophic illness as a liminal period.

### Celebrity makes a comeback on a comeback: Cojocaru**’**s kidney transplants

Typical organ failure accounts begin with the loss of the original organ and end with the (successful) transplant (examples include Etherington 1991; Hermans 2006; Klug & Jackson 2004). Cojocaru begins his account with the failure of his first transplant and ends it with the tenuous success of his second one. Uncertainty is a dominant theme throughout his account, unlike in Hermans's. His preface sharply contrasts an amusing dream he has been having (where various celebrities use glamour, hype and beauty products to console themselves about his death) with the ugly and unglamorous reality of kidney failure and a ‘carjacked’ life (Cojocaru 2007:xi).

His title, *Glamour, interrupted: How I became the best-dressed patient in Hollywood,* raises two crucial issues: glamour (for which he is famous) and interruption. The central struggle for Cojocaru in his book is how to reconcile his new damaged identity with his old professional persona of celebrity, particularly as his highly competitive fashion career is all about creating perfection.

Interruption is a form of disruption. It implies that life resumes after the interruption is over. An interruption is, furthermore, by definition, brief. Cojocaru treats this idea in a fairly complex way, because he had two transplants. After the first one, he tries desperately to escape persistent liminality and to return to what he knew before (success at this would be the end-point in a typical transplant narrative), but he cannot and he loses his kidney. He treats his second transplant differently.

In his first chapters he backtracks to how he discovered that polycystic kidney disease was destroying his kidneys and how he tried to deny this reality, preferring to lose himself in his high-powered job. Cojocaru spends a significant part of his book coming to terms with the idea of ESRD. He is at first so appalled by being flawed that he retreats into a state of denial that almost costs him his life. His greatest battle is to change his attitude to his new circumstances. ‘*I HAVE A DISEASE’* (Cojocaru 2007:10), he says, in capitals and in italics to show how overwhelming this news was for him.

One reason he rejects the idea of illness so strongly is because of what he does and where he lives. Success in Hollywood depends on external beauty and perfection. Surviving there is all about beautiful veneers. Illness is anything but glamorous, as he explains: ‘[*M*]y situation was unpleasant – a Hollywood euphemism for anything yucky, smelly, grotesque, or unattractive. If I went public with my disease, would the A-list turn its back on me?’ (Cojocaru 2007:16-17). Illness is a type of dirty secret, a hidden pollution, and Cojocaru tries very hard to keep it hidden.

When he is forced to accept his condition, he struggles to find a kidney and a friend eventually donates one to him. His language during this time is filled with stage and film imagery. He begins by ‘writing the script of [*his*] kidney transplant as a horror film’. Eventually, however, ‘[*t*]he stage was set’ he says for revealing the truth of his condition (Cojocaru 2007:26, 38). And so he reveals his flaw to the public.

This is cathartic and imagery of renewal becomes dominant. He is ‘drunk with a sense of rebirth’ (Cojocaru 2007:82). However, to have value in his world, he needs to be able to do his job and to be entertaining. He wants to show that ‘people with an illness could work and keep their sense of humour’ (Cojocaru 2007:42).

He tries and fails. His narrative becomes saturated by irony. Cojocaru the narrator is a knowing, ironic voice that has lived by Hollywood mores, but who mocks and reveals them. Cojocaru the narrated is an innocent who tries to live the Hollywood dream. This theme is an ironic reference to his first book, *Red carpet diaries: Confessions of a glamour boy* (2003), in which he gives readers glimpses into the extraordinary workings of behind-the-scenes Hollywood whilst describing the allure of glamour and his own star-struck awe at being admitted to the inner circle.

However his persistent denial about the seriousness of his condition and the finality of the losses he has sustained causes him to treat his new kidney roughly and he loses it. He is still trying to live the movie plot:

I was going to pick up right where I left off seven months earlier, put my life back on like a perfectly cut French suit. Everything would happen according to my master plan, no restrictions, no change of habits. It was suffer the transplant, go on *Oprah* and you're cured—almost like Oprah was a shaman who had given me the final healing I needed. (Cojocaru 2007:87)

His elderly mother donates the second kidney and this time, filled with guilt and fear, he tries to treat his transplant differently. Not only does he have to master a complex medical regime, but he has to re-evaluate his glamorous life in the face of bodily frailty and imperfection. In the course of his book he battles to come to terms with what has happened to him and his life and to accept that he can never go back to what he was before. At the end of his book he attempts to gain a type of closure and meaning from his experiences.

His last chapter describes his return to health in a different way from his first transplant: ‘The real pain comes afterwards, when you get back to your life’ (Cojocaru 2007:152). This time around he knows the life he knew before is over and his reference to ‘getting back’ to his life is ironic, because he knows he cannot go back to his life in the sense of returning to inhabit it. Instead he returns to assess and to change it.

Previously, on moving to Hollywood he had reinvented himself through fashion (Cojocaru 2003). Now he reconstructs himself through writing to obtain meaning from his circumstances. He has to accept a truth that has been almost unbearable: ‘Unlike the movie star I'd always been in my own mind, the real me was flawed’ (Cojocaru 2007:155). He ends his account with, ‘It didn't matter what I didn't have. Whatever I had *was* enough because I was alive’ (Cojocaru 2007:155, emphasis in the original). However, this in itself is an often-used movie plot.

He attains a degree of acceptance in coming to terms with not having what he had wanted. Nonetheless he is still keenly aware of his loss and his changed identity. Who he is now is not really unpacked. Perhaps the reality of being permanently changed is still unspeakable to him or maybe the aim of his book is to achieve coherence, not necessarily to explore below the attractive and entertaining surface. Maybe his identity is elusive to him because it is still liminal. Perhaps he views himself as work in progress. He is not who he was and yet, unlike then, he is now well. He is also between states, as he explains: ‘Disease doesn't go away, even when it's gone’. He feels separated from other people by his experience: ‘I wasn't like other people anymore’ (Cojocaru 2007:148, 151).

His writing style is journalistic, which allows him to name-drop and opens a door on a lifestyle that most of his readers would not experience. He makes it seem very exciting and desirable, but never allows the reader to imagine that the veneer of Hollywood is anything other than a veneer. One of his battles in his account is to accept that surface is not everything and that once that surface is damaged meaning has to be found elsewhere. This is what makes the ending of his account so elusive and the strategies of coherence so ironic.

Part of Cojocaru's professional reputation is built on irreverence for the structures of which he is part and this serves him well in his book. His final paragraph explains that ‘life is not a movie’ (Cojocaru 2007:155). He sets up his account as if it was one and then he undermines it with the untidiness of everyday life. Despite his irreverence he nonetheless constructs meaning in a fairly typical way: having to put aside the idea of bodily perfection, he seeks spiritual enlightenment, as in many Hollywood films. In the end he attains a familiar type of moral from his tale: riches and beauty are fleeting and do not necessarily bring happiness. Happiness must be found in other things. He concludes that he has a chance to live and that all the suffering has made his family a stronger unit. This is a type of redemption that allows him to escape the damnation of permanent physical imperfection.

His account is difficult to pin down in that he takes a narrative convention and twists it, but then, to some extent, untwists it. His account is uniquely his own, but also relevant, despite his extraordinary lifestyle, to many people. He creates a vivid picture of his circumstances, both health and professional, by using humour and juxtapositions that can be shocking. A favourite device of his is to use beauty and ugliness together and it emerges as a theme in his book that he traces back to his parents having fled Romania and having to present a façade of normalcy to survive. Amidst so much pampering and prettiness the medical experiences attain a type of brutality that is visceral. Hermans elides the brutality of such experiences, possibly because it could distract the reader from the redemptive ending.

Cojocaru uses humour as a defence mechanism. Humour and disavowal are the means through which he speaks about the unspeakable. This creates an ironic type of limbo for his understanding about changed identity, where the precise nature and extent of his understanding is unclear, because much of it is implicit and therefore not overtly stated. His account goes beyond a simple tale that is invested in recovery to one that considers to some extent what the cost of such a recovery and any type of redemption is.

### Making meaning from an unreliable renal redemption

Initially I found an account of Hollywood glamour and an account of spiritual redemption dissimilar. The most obvious difference was in their world views. The spiritual guru account by Hermans sees the world through a religious Christian lens and the celebrity one by Cojocaru is a Hollywood comeback narrative. But they do share certain qualities. These are the main ones.

The narrators are middle class residents of Western countries, so their biographies show middle class, Western concerns of atoning for perceived sins and the importance of being able to help oneself. There are other similarities. Both transplantees became seriously ill as adults, after being relatively well before then. Because of this, illness is indeed a disruption in their lives. For this reason life after transplant can to some extent be described as recovery or ‘being restored’ (Hermans 2006:106). ‘Normalcy’ for both transplantees is health. When the transplantees’ health is taken from them, the narrators perceive them as being in need of redemption. Cojocaru expresses this in so many words: ‘OH NO! IT'S MY FAULT!’ (Cojocaru 2007:112, capitals in the original). When his doctor manages to allay this fear, it re-emerges in throw-away remarks about ‘karmic fashion police’ and his not fitting his designer clothing (in which he vests so much of his public and private identities) anymore after his medication causes weight gain (Cojocaru 2007:113). He is no longer who he once was.

Not only this, but more significantly, both of them for different reasons describe illness as pollution. Hermans refers in her book to ‘toxins’ and the need for literal and figurative purification (Hermans 2006:55). From this, one can deduce that she sees real-life illness as ‘impure’. Cojocaru describes his kidneys as ‘rotting’ (Cojocaru 2007:18). Illness carries profound metaphorical and moral weight for both of them, as it does for many (Sontag 2001). Hermans and Cojocaru treat the ordeal of illness as a type of test of character, which helps them to derive meaning from it. However, if illness is seen as disruption as in Parsons's still-pervasive model (1951) and pollution, it can very easily be associated with sin or punishment. This could be why both books carry an element of a redemptive moral tale.

Ultimately both books are focused on recovery, but the recovered lives are irrevocably altered. Resistance to this change causes tension in both books. Part of the reason for this resistance lies in the type of account they tell. In many texts, both the parable and the Hollywood comeback narrative are redemptive narratives that control and order meaning through their structures and create an attractive surface that conceals tensions. These tales of renal recovery are no different. Both types of narrative require a belief in a certain type of agency: if you try hard enough and do things in the right combination you will be forgiven and allowed to return to ‘normalcy’. The Hollywood idea of a meaningful life and transcendent value has become a popular culture replacement for traditional religious beliefs. Whilst Cojocaru's book provides an ironic counterpoint to Hermans's, he still to some extent accepts that confessing is part of the process.

In Hermans's account redemption offers the promise of eternal life, although she is aware that her husband's transplant may not last forever. Cojocaru is similarly aware that his transplant's success cannot be guaranteed. Possibly because he has already lost a transplanted kidney he focuses more on this. Nonetheless both of them attempt to tie up the loose ends by imposing a redemptive narrative structure on their experiences.

Trying too hard at this can occlude liminality, but it does not, of course, eliminate it. Liminality's persistence can be found in the traces of perturbation that remain in the text in Hermans's uncertainty about her husband's future health and Cojocaru's unexpressed loss. It is also understood through both narrators’ recognition that the transplantees can never return to who they once were. A new life requires a new identity, but by the end of both books the transplantees still vacillate between being ill and well and between who they once were and who they hope to become.

## So what about me?

Transplantees always compare numbers. If we are not comparing our creatinine levels, blood pressure and medication dosages, we are comparing how long we were ill or how long we have had our kidneys. Especially the latter. I was ill for much longer than either transplantee I have been discussing (22 years) and now I have been well post-transplant for much longer than both added together (23 years).

‘Normalcy’ for both the other transplantees is health. When their health is taken from them, the narrators perceive a need of redemption. I perceive this as being strange. For me ESRD was just life. Not pleasant, but life as I knew it. I had grown up with it and with its attendant doctor visits, blood tests medication and diet. I often find it strange being well: it seems too simple, somehow. And also violent and chaotic. Everything is more intense. Good health really is rude. I still feel as if I am waiting for something to happen, perhaps the advent of normalcy. I hope I shall recognise it when I attain it.

The liminality I experience has a different quality now than it did, say, 15 years ago. Back then it was the heady anticipation of increased levels of well-being. My health improved steadily for the first five years. Each time I thought I was as well as any human being had a right to expect, my creatinine levels would drop or my medications (and hence their side-effects) would be reduced and I would improve. Every morning I awoke for the first eight years post-transplant I was filled with awe and gratitude for my new and bounteous health that I feared might disappear without warning.

Somewhere in the decade that followed I became used to my bounty. These days when I wake up in the mornings the first thing on my mind is usually much more prosaic: a concern about work or wondering about whether I have time to doze a little longer. The question on my lips these days no longer concerns existential matters, but rather whether I should have muffins or muesli for breakfast, Earl Grey tea or Darjeeling. Friends tell me that this is what being well is. Apparently you expect health and are not surprised by it.

And yet I am still in a liminal space. I still go for doctor's visits, take my medications, and have blood tests. I should keep to a diet better, but here again my liminality raises its head. When I see previously forbidden foods (the list was immensely long as renal diets are very restrictive) I cannot help eating as much as I possibly can, as if I may not be able to have more tomorrow. All the while, I mentally weigh the amount of renal-unfriendly protein, salt, cholesterol and potassium, thinking, ‘bad, bad, bad’. I recently missed an outing with friends and my chief regret was that they had had prawns for lunch and I had not, although I can buy prawns and eat them whenever I want.

Not too long ago my doctor suggested I lower my cortisone dose to a tablet every second day. I have taken daily medication since I was seven and have been responsible for it since I was nine. I claim my medications have the same emotional charge as brushing my teeth or taking a vitamin supplement. But this is not so. Lowering my dose by 5 mg filled me with such superstitious dread that every slight fluctuation in temperature, every bit of fatigue seemed the harbinger of kidney failure or rejection. I took my pulse, my blood pressure, my temperature at every hint of a change in my physiology. I palpated my kidney. I emailed all my friends about it. I barely slept. Despite my alarm, my condition remained stable. I was surprised.

And so I continue, a patient and yet not a patient, well and yet not, able-bodied and yet disabled, treated and not cured, healthier than I have ever been, thanks to a part of a stranger's body that somehow has managed to live in mine for over 20 years. Like Hans, I do not know how long my kidney will last. I do not know how long I will last either. Like Cojocaru I know that whatever I have is enough because I am alive. I suspect this has prevented me from reaching for what I want in life. That and my overwhelming instinct to save my energy and protect my health at all costs.

During my PhD research I simultaneously worked and studied full time and I managed this longer than I thought possible. One year, however, I became quite run down and towards the end of the year fell seriously ill with complications resulting from chicken pox. My kidney survived that too. It was (I think) the only organ in my body not to be affected. (It could be that my donor had chicken pox, but I shall never be able to confirm that because my kidney is a non-related, non-living transplant.) That surprised me, but not nearly as much as the ease with which I adapted to my lengthy sick role again or how at home I felt in the hospital although I was hospitalised in a foreign country and had not been admitted to hospital since 1992, a year after transplant.

Only much later when talking to friends who were concerned that I had been lonely (I had not been), did I realise that I had not been at all concerned about being in an isolation ward. True, I was very ill, but I was also unperturbed by my captivity. I had no television and so I amused myself playing with the electric bed adjuster. Last time I was in hospital we had had to adjust our beds with a pedal and the novelty of doing this with the flick of a button diverted me for many hours. I was quite content to be fed and examined, sometimes simultaneously, for days. It made me feel safe. That perturbs me now, because all the well people I know shudder at the thought and it reminds me that even after all these years I am not really one of them.

I have been writing about my journey at last, because enough time has passed to give me the necessary distance and to make the task less overwhelming. In writing my own book-length account, I have been struck by how difficult it is to make a narrative that is both meaningful to me and a true reflection of what happened. It is easy to fall into conventional patterns of meaning-making and I find them ultimately unsatisfying. When one writes about one's experiences, one is creating meaning from one's suffering. But does this rob one's life account of complexity? The idea of suffering redeeming one, having a purpose or making one a better person might be attractive, but life is too messy for that and liminality too complex. I continue to wrestle with this.

I wonder if there is a period after transplant when one stops being liminal. Possibly life itself is a liminal period for everyone and yet this liminality is only recognised when something extraordinary happens. For many of my fellow transplantees liminality is something that they only become aware of when faced with mortality. They remember normal good health by contrast as being a stable period. For me health itself is unstable and ever changing. Living this way feels liminal to me, a constant state of flux and violently surging chemical processes. I did not recognise hunger when I first felt it after transplant. I thought the stomach pains I got at lunch time were a sign of dreadful complications or side-effects to my new medications. It still startles me and my reflex response is to treat it as if it were a problem that must be medicated with food. But, despite all the complexities and uncertainties of my new life (as I still think of it), I do not long for the ending of liminality as some of my fellow transplantees may, so much as the continuation of it.

## Conclusion

Whilst the impetus for writing Cojocaru's and Herman's accounts was different for the narrators, ironically both the parable and the Hollywood drama are redemptive narratives that attempt to control and order meaning to create coherence and tidy up the messiness of lingering liminality. When one writes about one's life, one is creating meaning. The audience expects it. However, a danger I see in an account that suggests life is somehow better or more profound after a catastrophic event, is that it positions the narrator as a guru who has unique access to the truth and makes the account a moral tale. To some extent, both accounts I have discussed do this. This idea of suffering redeeming one, having a purpose or making one a better person might be attractive, but life is too contingent for that.

I too want redemption, but all these years after transplant my perspective has changed. I have come to see that although society may expect me to emerge from my liminal stage in a new role, improved and whole as Parsons (1951) described, this is not really possible for someone in my state. Paradoxically I also see that my experience is normal. I do not need to seek redemption or to try to escape liminality. I think I would be disquieted if the questions in my life were suddenly resolved. I would not know what to do with myself. I feel ambivalent about liminality, but I suppose one would. It is, after all, such an ambivalent state. Ironically, at this point in my life post-transplant, I seem inadvertently to have achieved some shaky sort of closure after all. I take comfort in the fact that if the past is anything to go by this is likely to change in the future. Another two or so decades down the line and I am certain my views on the matter will have changed again. But that is what chronic illness is – the long distance run. If you live with something your whole life your demeanour towards it will change.

From experience I can assure you that it is frightening exploring your own version of persistent liminality. You might fear that you are living in-between worlds because you are doing something wrong. Because of this, I think we need more research on the different types and stages of liminality after catastrophic illness, especially a kidney transplant.

## Footnotes

1 I use this comparison because it is one that other researchers (see above) use in the literature. Other types of organ failure can be seen (wrongly) as more life-threatening than kidney failure. Possibly this is because one can receive an entire kidney from a living donor and there is the back-up of dialysis for people with access to resources. In addition, people may find it easier to understand heart disease than kidney disease, as we are aware of our heart-beat and of any changes in it. The heart's symbolic value is immense: it is the site of love, life and energy. Kidneys are quite invisible by contrast.
